# Comparison of Physical Activity, Dual-Task Performance, and Cognitive Skills Between Problematic Video Game Players and Control Subjects

**DOI:** 10.7759/cureus.31073

**Published:** 2022-11-04

**Authors:** Cagtay Maden, Begümhan Turhan, Erkin O Sarı, Kezban Bayramlar

**Affiliations:** 1 Physical Therapy, Gaziantep Islamic Science and Technology University, Gaziantep, TUR; 2 Department of Anatomy, Faculty of Medicine, Baskent University, Ankara, TUR; 3 Physical Therapy, Hasan Kalyoncu University, Gaziantep, TUR

**Keywords:** physical activity, gaming disorder(gd), cognitive skills, dual task, problematic online gaming

## Abstract

Introduction: This study aimed to compare the dual-task and cognitive skills of problematic video gamers and non-problematic video gamers based on the fact that cognitive skills (e.g., inhibition, decision-making, attentional control, time perception) and dual-task performance may be positively affected in individuals who play games.

Methods: The study was conducted on 62 individuals. The study group (n=33) consisted of individuals who played games, and the control group (n=28) consisted of non-gamers. Their scores on exercise benefits and barriers perception, cognitive performance, cognitive skills, and dual-task performances were measured. The Montreal Cognitive Assessment (MoCA) scale was used to evaluate cognitive function, and the Trail Making Test (TMT) was used to determine attention, speed, and better motor performance. The Stroop test was used to evaluate executive functioning.

Results: It was determined that the problematic game players group was faster at the Stroop 1 and Stroop 2 test times (respectively p=0.020, p=0.005). In the comparison of dual task-cognitive 10-meter walking) test times of both groups, it was seen that individuals in the problematic game players group were faster than the control subjects (p=0.044).

Conclusion: It can be said that playing digital games improves the cognitive dual-task cost (DTC) and the executive functioning of individuals.

## Introduction

Cognition is defined as neural processes underlying many functions and includes memory, social interactions, emotions, empathy, and visual and motor functions [1]. Briefly, it is the process of the brain being involved in understanding and functioning in the external environment. The skills involved in this process are called "cognitive skills" (e.g., inhibition, decision-making, attentional control, time perception). In addition to basic skills such as memory, remembering, and orientation, functions such as problem-solving, perception, attentional capacities, response speed, decision-making, etc. are also among our cognitive skills [2]. We need these skills to carry out any task, from the simplest to the most complex functions [3]. Dual-tasking, which requires a person to perform two tasks at the same time, is known as a neurophysiological process and is related to the subcortical components of the nervous system (basal ganglia, cerebellum, vestibular nucleus, red nucleus, substantia nigra, subthalamic nucleus, reticular formation) [4]. When two tasks are performed simultaneously, “attention capacity” must be apportioned effectively. The difficulty of these tasks, or how tasks are prioritized, will influence how attention will be partitioned. However, if the combined difficulty of the tasks requires excessive attention, then interference between tasks could occur. That is, the quality of performance of both tasks could decrease, or one task could be performed in preference to the other [5]. Cognitive skills and dual-task cost (DTC) can be positively affected by daily routine activities, physical activity, and video gaming. It is known that especially physical activity improves dual tasks and is affected by physical activity level [6]. Several studies have shown that video game playing activity can improve cognitive functions such as visual attention, cognitive control, visual short-term memory, and general neural processing speed. Previous research also has shown that video gaming alters the structure and functions of the brain. The hippocampus, cerebellum, frontal eye field, thalamus, gyrus lingualis, temporo-parietal junction, and lateral occipital cortex have been reported as anatomical structures with particular neural changes [7]. 

Also, there are studies that report that video game experience leads gamers to possess perceptual and cognitive skills far beyond those observed in non-gamers [8,9]. Furthermore, some of the studies found that playing action video games had no effect on problematic video gameplay. Problematic video gameplay may not have a detrimental or beneficial effect on cognitive performance, although this is difficult to ascertain. More research is therefore needed. In our study, we aimed to compare the dual-task and cognitive skills of problematic video gamers and non-gamers, based on the fact that cognitive skills and dual-task performance may be positively affected in individuals who play video games.

## Materials and methods

Participants and study design

**Figure 1:**
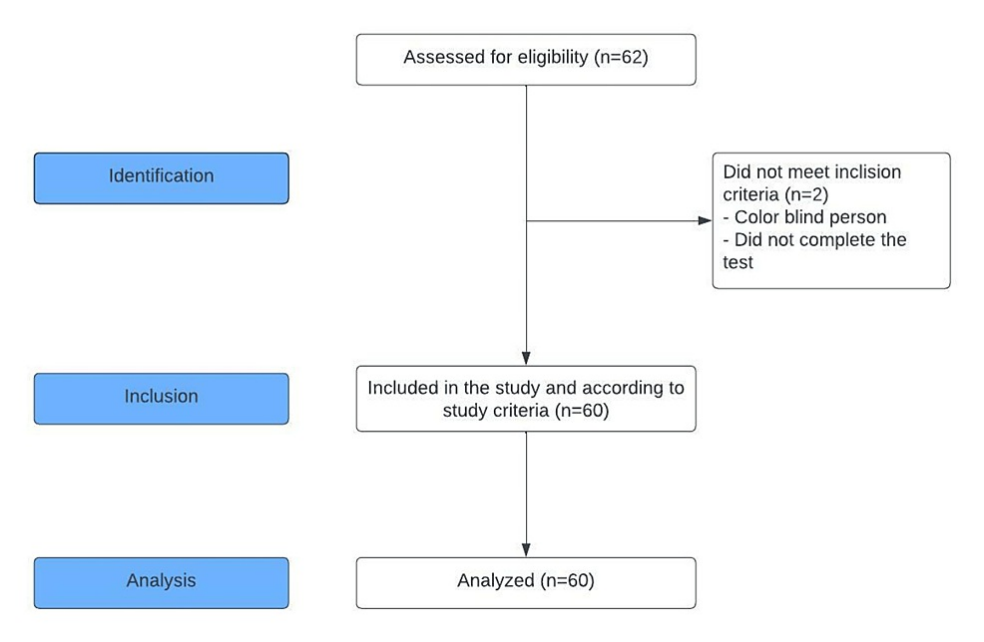
Strengthening the Reporting of Observational Studies in Epidemiology (STROBE) flow-chart diagram

In this cross-sectional study, selected male students (according to their problematic or non-problematic video gaming characteristics) from Hasan Kalyoncu University aged between 18-24 were evaluated. As per the literature, it is expected that problematic video gaming is generally associated with the male gender [10]. Disorder of control over gaming activity is described as a pattern of "digital gaming" or "video game" behavior characterized by the increased priority given to play over other activities to the extent that play takes precedence over other interests and daily activities. Despite its negative consequences, it is stated as a continuation of the video game [11]. A total of 62 individuals participated in the study. The study was conducted between January 2020-January 2021. The study group consisted of individuals who played video games (90% first-person shooter games, 10% multiple online battle arena games) for a minimum of two hours or more every day in the last 12 months [11]. The other group is made up of non-gamers who have never played video games on any device (phones, computers, etc.) in the last year. All of the individuals were self-reportedly healthy and had no mental or medical issues that should preclude them from participation, which is expected given that the participants were presumably normal. Two individuals, who were color-blind and not cooperative during evaluation tests, were excluded from the study (Figure 1).

Ethical approval

The study protocol was approved by the Non-Interventional Research of the Faculty of Health Sciences, Hasan Kalyoncu University on December 3, 2019 (Protocol no: 2019/112). Informed consent was obtained from all participants and registered on ClinicialTrials.gov (ClinicialTrials.gov ID: NCT04860557). The study was conducted according to the Declaration of Helsinki.

Measurement tools

Demographic information and physical activity levels of the participants were noted. To learn the physical activity levels were important because dual-tasking could be affected due to the high physical activity levels [6]​​​​​.​ Their scores on exercise benefits and barriers perception, cognitive performance, cognitive skills, and dual-task performances were measured.

International Physical Activity Questionnaire (IPAQ)

The IPAQ was used to evaluate physical activity levels. This questionnaire determines the duration and frequency of different types of activities in the last seven days. It consists of four sections (high-intensity activity, moderate-intensity activity, walking, and sitting categories) and has a total of seven questions. Calculation of the total score in the questionnaire includes total time and days of walking and sitting activities, and moderate and high-intensity physical activity [12].

Exercise Benefits and Barriers Scale

This scale comprises 43 items, and studies the level of individuals’ perception of benefits/barriers of physical activity with a 4-point Likert Scale (4=Strongly agree to 1=Strongly disagree). On the barrier scale, scores between 14-56 can be obtained, and a high score means there are many barriers to doing exercise. The benefits scale can be scored between 29-116, and a high score means that the individual has a high belief in terms of the benefits of exercise [13].

*Montreal Cognitive Assessment (MoCA) *Scale

The MoCA scale was used to evaluate cognitive function. This inventory was developed as a rapid screening test for mild cognitive impairment. The MoCA evaluates different cognitive functions. These include attention and concentration, executive functioning, memory, language, visual configuration skills, abstract thinking, calculation, and orientation. The highest score on the test is 30. Accordingly, a score of 21 or above is considered normal [14].

Trail Making Test (TMT)

The TMT was used to evaluate cognitive performances. This test is a visual screening test, and evaluates planning, organization, motor function, visual-motor perception, abstract thinking, and reaction limitation. Test success requires attention, speed, and better motor performance. The test has two sections namely, A (TMT-A) and B (TMT-B). Section A consists of only numbers. The individual is asked to draw numbers starting from 1 to 25. Section B, which is more complex, consists of both numbers and letters. In section B, the participant is asked to combine a number and a letter in order. The TMT-A provides insight into cognitive processing skills (psychomotor speed, processing speed, and visuospatial ability), while TMT-B provides information about executive functions. Completion time is recorded in both TMT-A and TMT-B [15].

Stroop Test

The Stroop test is used to evaluate executive functioning. The Stroop color-word test is a widely known and robust measure of selective attention and interference. The Stroop test measures the ability to inhibit automatic response tendencies [16]. In our study, the Stroop-TBAG (Stroop test Turkish version) form was used. Three different measurements were recorded during the test. The Stroop test consisted of three stages. In Stroop 1 (S1), participants were asked to identify the colors of the figures, and the time taken for identification was recorded. In the Stroop 2 (S2) stage, participants were asked to identify the colors of the different words, and the duration of the response time was recorded. In the third stage, the time taken to distinguish between the colors represented in Stroop 3 (S3) and the names "yellow, red, green, and blue" printed in different colors (E.g., the word “blue” written in yellow color) were recorded.

Dual Task

Dual-task is the concurrent performance of two independent single tasks that have distinct goals. In our study, the '10-Meter Walk Test' (10-MWT) and the 'Timed Up and Go Test' (TUG) were used to assess dual-task. In the 10-MWT, individuals walk at their normal walking speed and the time is recorded in seconds [17]. The TUG measures the time it takes a subject to stand up from an armchair, walk a distance of 3 m, turn, walk back to the chair, and sit down. The elapsed time is recorded in seconds [18]. During these two tests, a secondary motor task (90° shoulder flexion, anatomical position, 90° shoulder abduction, anatomical position, 180° shoulder flexion, anatomical position, respectively) and a secondary cognitive task (naming words beginning with “K” in TUG, and naming words beginning with “N” in 10-MWT) were performed to evaluate dual-task outcomes. Finally, 10-MWT, 10-MWT motor dual-task duration, 10-MWT cognitive dual-task duration, TUG time, TUG motor dual-task duration, and TUG cognitive dual-task duration were recorded.

Data analysis

For statistical evaluation, SPSS for Windows, version 25.0 (IBM Corp., Armonk, NY) was used. G*power analysis was used to determine the sample size. The minimum number of subjects required for the difference between the dual-task cost variable between the two groups to be significant was determined as 26 (α=0.05 and test power=0.80). The distribution of data was evaluated by the Kolmogorov-Smirnov test. All parametric (continuous variables) results were expressed as means and standard deviations for each group. Data comparisons between groups were analyzed with an independent sample t-test. A level of significance of p<0.05 was accepted for this study. DTC, the change between single and dual-task performance was calculated by using the formula "DTC = [single task time - dual task time] / single task time × 100" [19].

## Results

Comparisons of baseline characteristics

The average age of all subjects was 22.5±1.3 years. Ages were similar across the groups (p=0.696). The sample consisted of male individuals. The physical activity level (MET-min/per week) of the non-gamers was higher than the problematic game players group (p=0.047). The daily sitting time (sedentary time) was more in the problematic game players group (p=0.017). The average age and physical activity levels of the groups are given in Table 1.

**Table 1:** Physical activity levels and the exercise perception of the subjects *p<0.05 Independent samples t-test IPAQ: International Physical Activity Questionnaire

	Problematic Game Players (n=33) (X±SS)	Non-gamers (n=28) (X±SS)	T	P
IPAQ MET (sec/week)	1210.9±683	1738.3±1248.5	-1.978	0.047*
Sedantary Time (sec)	418±121.5	332.7±123.8	2.473	0.017*
Exercise barriers scores	29.1±7.1	28.7±7.5	0.229	0.820
Exercise benefit scores	55.6±12	52.6±15.2	0.883	0.381

Baseline outcomes of the subjects

When the cognitive scores and cognitive levels of the groups were compared, it was determined that the problematic game players group was faster than non-gamers in the Stroop 1 and Stroop 2 test times (p=0.020, p=0.005 respectively). As shown in Table 2, there were no significant differences between the groups in terms of MoCA, TMT-A, TMT-B, TUG (normal, cognitive, motor), and 10- MWT (normal, cognitive, motor).

**Table 2:** Comparison of the groups in terms of cognitive skills and cognitive levels of the groups *p<0.05 Independent samples t-test MoCA: Montreal Cognitive Assessment Scale, TMT: Trail Making Test, TUG: Timed Up and Go Test

	Problematic Game Players (n=33) (X±SS)	Non-gamers (n=28) (X±SS)	T	P
MoCA Score	27.5±1.6	26.9±2	1.317	0.174
TMT-A (sec)	18±4.6	19.6±5.6	-1.215	0.229
TMT-B (sec)	39.2±8.4	43.3±12	-1.505	0.138
Stroop 1 (sec)	10.3±1.6	11.5±2,1	-2.402	0.020*
Stroop 2 (sec)	12±1.6	13.7±2,4	-2.919	0.005*
Stroop 3 (sec)	17.9±3	19.1±2.9	-1.504	0.138
TUG normal (sec)	7.5±0.9	7.4±0.6	0.332	0.741
TUG cognitive (sec)	9.1±1.3	8.9±1.3	0.558	0.579
TUG motor (sec)	8.6±1.1	9±0.9	-1.132	0.262
10-meter normal (sec)	7.7±0.8	7.6±0.8	0.107	0.915
10-meter cognitive (sec)	9.5±1.4	10.1±1.6	-1.359	0.175
10-meter motor (sec)	8.5±0.9	9.1±1.3	-1.704	0.094

In the comparison of dual task-cost cognitive (10 MWT) times of both groups, it was seen that problematic game players group subjects were faster than non-gamers (p=0.044). As shown in Table 3, there was no difference between the groups in terms of time spent on other tests.

**Table 3:** Comparison of the groups in terms of dual-task *p<0.05 Independent samples t-test TUG: Timed Up and Go Test, DTC: Dual-task cost

	Problematic Game Players (n=33) (X ± SS)	Non-gamers (n=28) (X ± SS)	T	P
TUG cognitive DTC	22.7±14.4	21.2±16.1	-0.364	0.714
TUG motor DTC	17.3±18.4	21.6±11.4	1.071	0.311
10 metre cognitive DTC	23.9±13.7	31.9±16	2.057	0.044*
10 meter motor DTC	12.1±14.3	18.1±11.3	1.759	0.091

## Discussion

The current findings of our study, which is based on the fact individuals who play video games may be affected by cognitive skills and dual-task performance, were that the physical activity levels of the non-gamers group were higher than that of the problematic game players group. Also, we determined that the cognitive DTC-10 MWT, Stroop 1 and 2 tests’ performances of the individuals in the problematic game players group were faster than the non-gamers. MoCA and TMT values were found to be similar in both groups. There was no precedence found between the two groups in TUG cognitive DTC, TUG motor DTC, and 10-meter motor DTC values.

Based on previous research, it is expected that problematic video gaming is associated with the male gender in the gaming population of the world [10]. Generally, female individuals are not encouraged to play video games [20]. Because all the individuals in our study were males, gender-related differences could not be revealed by this study.

Many studies in the literature determined that video game players spend excessive time with digital games and they have low physical activity levels [11]. Henchoz et al. indicated a reciprocal causality between video gaming disorder and the level of physical activity [21]. In particular, this shows that individuals who spend time playing video games spend less time on physical activity. In our study, the fact that the physical activities of individuals who do not play video games are higher than those who play video games supports this situation. Also in our study, the sedentary time of the problematic game players group was more than the non-gamers group. Thus, we can say that the problematic game players’ lifestyles caused low physical activity tendency, or oppositely low physical activity levels may be the cause of problematic video gaming. Girgin and Okudan stated that higher physical activity levels were associated with an increase in the perception of exercise benefits and a decrease in the perception of barriers to exercise [22]. In our study, both groups thought that exercise was beneficial, and the evaluation compared the difficulties of exercising at similar levels. However, the physical activity levels of the video game players group were found to be lower than the non-gamers group. This may be due to the problematic video gamers' preference for playing video games as a leisure activity.

It has been reported that digital gaming affects the cognitive capacity of gamers [23]. Lopez-Fernandez et al. stated that video games have benefits in terms of enhancing cognitive, social, and physical abilities [20]. On the other hand, some studies demonstrate that the pathological or excessive use of video games leads to more negative consequences on cognitive processes [9]. These complicated results, which researchers attribute to various factors, are still being investigated. Similar to some studies in the literature, some of the cognitive measurements of the problematic gamers group in our study are better than the non-gamers group.

In our study, the score of both groups were higher than the MoCA cut-off value of “23” [24]. This means that the cognitive performances of the participants in our study were within the normal limits, and did not differ from each other. TMT and Stroop tests have been used in previous studies to evaluate the cognitive skills of video players [25]. Both groups of our study had similar scores in the TMT and this may be due to the fact that our sample was university students. There are many activities related to visual scanning, planning, and organization in student life (e.g., reading books, reading notes and presentations, etc.).

In the literature, the results of the studies in which a cognitive assessment was performed on gamers with the Stroop test are variable. In some studies, Stroop test results were better than the control group [26], while some others were the opposite. This situation can be associated with the level of problematic gaming. Stroop test results were found to be lower in the individuals who were problematic gamers at the addiction level compared to the control group [27]. In our study, the individuals in the problematic gamers group were not at the addiction level. We think the difference between groups in Stroop tests could be related to the type of games they play. Steenbergen et al. emphasized that video gaming and especially "first-person shooting" games improve cognitive performance [28]. It should be noted that the game type can affect the Stroop test values. It can also be attributed to the fact that this test examines the frontal lobe in evaluating the cognitive level and makes precise measurements of visual performance.

The validity and reliability of the TUG test, that we used in the dual-task evaluation, were reported in a study conducted with healthy young individuals [29]. The fact that there was no difference in TUG scores between the groups in our sample may be related to the insufficient test distance of the TUG test in order to evaluate the dual-task effect. In this study, the 10-MWT distance was longer than the TUG test's distance. In our results, 10-MWT (cognitive DTC) scores were different between groups. We think that the difference between the groups in the dual-task measurement in the 10-MWT was related to the better determination of DTC over a longer distance. Also, we think that using both eye-hand coordination and using more cognitive skills at the same time during video games improves dual tasks. The fact that the motor skills of dual tasks are similar in individuals who play problematic games compared to individuals who do not play can be explained by the use of only hand movements in video games and not using other gross movements. Howell et al. stated that physical activity improves DTC [6]. Ogawa et al. stated that video-based exercise training has benefits on dual-task performance and cognition [30]. Studies reporting the different positive effects of playing digital games in the literature have also reported that there may also be positive effects on cognitive and dual tasks. In our study, although the physical activity levels of the problematic game players were low, the fact that they had better cognitive dual-task in the 10-MWT than the non-gamers group could be explained by their playing of digital games. ​It can be said that playing digital games improves the cognitive DTC and executive functioning of individuals. More studies are needed on the cognitive status of problematic gamers.

There were a few limitations in the current study. First, the psychoneurological diseases were recorded according to the personal statements. Second, gender differences could not be analyzed as there were no female individuals with gaming disorders. In addition, the types of video games were ignored. We also conducted the study during the Covid-19 pandemic. Due to pandemic conditions, individuals' physical activity levels might be lower, and it should be taken into account that the playing time might be longer.

## Conclusions

In conclusion, digital games can provide benefits in the daily life and educational life of individuals in terms of dual-task performance and cognitive functions. However, playing video games can cause lower physical activity levels. To address this problem, for example, exergames can be used to improve both the physical activity levels and dual-task cost in these individuals. Future studies on problematic gamers can be expanded by examining the changes in brain structure and brain function. Future research also needs to take into account individual differences.
